# ICAM signaling rewires the inflammatory communication network in pediatric gout

**DOI:** 10.3389/fimmu.2026.1871249

**Published:** 2026-06-29

**Authors:** Shengyou Yu, Wei Lu, Dingxuan Zhang, Qi Ren

**Affiliations:** 1 Department of Pediatrics,Guangzhou First People’s Hospital, South China University of Technology, Guangzhou, Guangdong, China; 2 Department of Radiology, Guangzhou First People’s Hospital, Guangzhou Medical University, Guangzhou, Guangdong, China; 3 Department of Rheumatology and Immunology, Guangzhou Women and Children’s Medical Center, Guangzhou Medical University, Guangzhou, Guangdong, China

**Keywords:** cell-cell communication, ICAM1, monocytes, pediatric gout, single-cell RNA sequencing

## Abstract

**Introduction:**

The immune regulatory characteristics and core inflammatory signaling networks of pediatric gout have not been fully elucidated. The prevailing view of generalized immune hyperactivation cannot precisely explain the unique immune pathological features of pediatric gout, highlighting an urgent research gap to be addressed.

**Methods:**

To clarify the immune regulatory mechanism of pediatric gout, we performed single-cell transcriptomic profiling and systematic cell-cell interaction analysis on peripheral blood samples collected from pediatric gout patients and healthy controls. We comprehensively compared the intercellular communication networks, signaling pathway activities, and molecular expression patterns between the two groups, and focused on the differential inflammatory signaling axes dominating pediatric gout pathogenesis.

**Results:**

Different from the traditional view of global immune hyperactivation, pediatric gout exhibited a highly selective remodeling of circulating immune networks. The ICAM signaling pathway was identified as the most dominant and disease-specific inflammatory axis in pediatric gout. In the immune communication network, platelets served as the major signal-emitting hubs with significantly enhanced signaling activity in patients, while CD14^+^ monocytes acted as the primary signal recipients. Selective upregulation of ICAM1 was detected in CD14^+^ monocytes of pediatric gout patients, which endowed monocytes with strengthened signal -receiving capacity. Notably, other classic immune pathways showed no significant differences between patients and healthy controls.

**Discussion:**

This study demonstrates that pediatric gout is characterized by directional and selective immune crosstalk rather than universal immune activation. The ICAM-centered inflammatory interactome is the core driver of immune network remodeling in pediatric gout. These findings complement and advance previous single-cell research on adult gout, and identify the ICAM signaling pathway as a novel and promising therapeutic target for the precise clinical intervention of pediatric gout.

## Introduction

Gout is initiated by monosodium urate (MSU) deposition but sustained by a multicellular inflammatory program that includes NLRP3/IL-1β activation, TLR-MyD88-NF-κB signaling, neutrophil extracellular traps, and ICAM1-associated adhesive responses (1–3). Current most mechanistic models have been built from adult disease. Although adult gout has been examined in several transcriptomic and immunologic studies, the communication architecture of pediatric gout remains largely unexplored. In particular, it is unknown whether pediatric gout reflects a generalized amplification of immune signaling or a selective rewiring of specific inflammatory pathways and cell-cell interactions. Resolving this distinction is important, because broad immune activation and pathway-selective network remodeling imply fundamentally different models of disease organization and suggest different therapeutic strategies.

Recent single-cell studies in adult gout have already moved the field beyond bulk inflammatory markers. Acute gout profiling in blood and synovial fluid showed enrichment of classical monocytes in peripheral blood together with major inflammatory redistribution across compartments (4). Paired flare-remission analyses then demonstrated that gout is accompanied by altered monocyte differentiation, HLA-DQA1hi nonclassical monocytes, Treg-linked resolution programs, and remodeled monocyte-T-cell communication (5). Integrative transcriptomic and Mendelian-randomization analyses further strengthened the view that classical monocytes and NLRP3/IER3-associated programs occupy a central position in gout flare biology (6, 7), while multi-omics genetic studies indicate that gout is also shaped by broader cell-state-specific regulatory programs rather than by urate concentration alone (8). ICAM1 is especially relevant in this context because it sits at the interface of inflammatory signaling and physical cell-cell engagement. ICAM1 transcription is inducible through NF-κB, C/EBP, AP-1, MAPK, and JAK-STAT pathways, making it a convergence node for inflammatory stress rather than a passive surface marker (9, 10). Experimental hyperuricemia similarly links increased ICAM-1 expression to NF-κB activation and inflammatory organ injury (11, 12). These data argue that ICAM1 should be interrogated not only as an endothelial injury marker, but also as a candidate organizer of inflammatory circuitry.

In this study, All enrolled pediatric gout patients were diagnosed as primary gout without inherited metabolic genetic syndromes, congenital immune deficiencies, or other underlying monogenic diseases. No case was classified as secondary gout induced by renal impairment, hematological disorders or long-term drug exposure. we integrated peripheral blood single-cell RNA sequencing with systematic cell-cell communication inference to define the inflammatory interactome of pediatric gout. We found that pediatric gout is not characterized by a diffuse increase in all immune interactions, but by a potential selective rewiring of the circulating immune communication network, which needs further validation in larger cohorts. This remodeling is dominated by a disease-specific gain in ICAM signaling, accompanied by coordinated activation of SN signaling, functional conversion of platelets into a major outgoing signaling hub, and preferential signal reception by CD14+ monocytes. At the molecular level, selective enrichment of ICAM1 in CD14+ monocytes provides a mechanistic basis for this communication asymmetry. Together, these findings identify an ICAM-centered inflammatory signaling axis as a structural organizer of disease propagation and reposition pediatric gout as a disorder of aberrant immune communication superimposed on canonical urate-driven inflammation.

## Materials and methods

### Patient recruitment

In this study, All pediatric gout patients and healthy children were recruited from the Department of pediatric, Guangzhou First People’s Hospital,Guangzhou, China,during the recruitment period from January 2024 to August 2025.Healthy children were age- and gender-matched physical examination children without metabolic, autoimmune or infectious diseases. children with gout fulflled the classifcation criteria of gouty arthritis (13). We collected basic patient information including age, uric acid level, glomerular fltration rate, and disease duration. Peripheral blood was obtained from three children with gout and three normal children, and single‐cell suspension was immediately prepared and subjected to scRNA‐seq analysis. All children and their parents consented to having their peripheral blood used for research purposes, and the study was approved by the Research Ethics Committee of Guangzhou First People’s Hospital, South China University of Technology, China (K-2024-129-02).

### Data analysis

Raw sequencing data were first processed and evaluated for quality control prior to downstream analyses. Briefly, FastQC (v0.11.9) was used to perform basic quality statistics and examine the overall quality of raw sequencing reads, covering base quality distribution, GC content, and potential adapter contamination. Raw FASTQ sequences generated from the Illumina sequencing platform were preprocessed using Trimmomatic software (v0.39) with the following filtering parameters: LEADING: 3, TRAILING: 3, SLIDINGWINDOW: 4:15, and MINLEN: 36. Only reads that met these stringent filtering criteria were retained as clean reads for subsequent bioinformatic analyses. FastQC was applied again to verify the improved quality of cleaned reads, ensuring the integrity and reliability of the final dataset. All samples exhibited an average sequencing depth of more than 50,000 reads per cell, which satisfied the standard criteria for single-cell immune profiling based on the 10x Genomics platform.

Clean high-quality reads were aligned to the human reference genome GRCh38 (2020-A version) using Cell Ranger (v6.1.2) integrated with the STAR splice-aware alignment algorithm. Mapped reads were systematically categorized into exonic, intronic, and intergenic regions according to genome annotation GTF files. Specifically, reads with over 50% overlap with exonic sequences were defined as exonic reads; non-exonic reads overlapping with intronic regions were classified as intronic reads; and the remaining reads were assigned to intergenic regions. For reads mapped to multiple loci containing both exonic and non-exonic regions, exonic alignment was prioritized, and such reads were assigned a high-confidence mapping quality (MAPQ = 255). Only reads confidently and uniquely mapped to the annotated transcriptome were retained for subsequent unique molecular identifier (UMI) quantification. To distinguish genuine single cells from background noise and artifacts including empty droplets and ambient RNA contamination, a sample-specific dynamic threshold strategy based on UMI count distribution was applied. The expected cell number was preset to 3000, and valid cell barcodes were defined as those with UMI counts exceeding one-tenth of the 99th percentile UMI value of top-ranked barcodes. This filtering process was performed independently for each library and reference genome. In total, 16,728 high-quality single cells were acquired from the six enrolled samples after rigorous filtering.

To eliminate technical batch variation across different samples and preserve authentic biological heterogeneity, batch effect correction and data integration were performed in the Seurat (v4.3.0) analytical pipeline using the Harmony algorithm (R package, v1.0). The top 30 principal components were adopted for cross-sample alignment between patient and control datasets, which efficiently minimized batch-derived biases while retaining intrinsic biological signals.

For dimensionality reduction and cell clustering analysis, normalized gene expression matrices were firstly subjected to principal component analysis (PCA) to extract core transcriptional features, transforming the original cell−gene matrix into a low−dimensional cell−component matrix. The pipeline adopted an optimized Python implementation of the implicitly restarted Lanczos bidiagonalization algorithm (IRLBA) to reduce memory consumption during PCA calculation (14). The optimal top 30 principal components were determined based on elbow plot and JackStraw statistical analyses and used for subsequent unsupervised cell clustering. For two-dimensional data visualization, PCA-reduced data were processed using t-distributed stochastic neighbor embedding (t-SNE), a classic nonlinear dimensionality reduction method for single-cell transcriptome visualization (15). The t-SNE implementation was optimized with a fixed pseudo-random number generator seed for full reproducibility and compiled with restricted output dimensions to improve operational efficiency, with the perplexity value set to 30 in this study. Cell clustering was conducted using the Louvain graph-based algorithm at a resolution of 0.8, which balanced clustering resolution and biological interpretability and successfully grouped cells with highly consistent gene expression patterns.

Cell type annotation was performed according to well-recognized canonical immune marker genes, including CD3D for T cells, CD14 for monocytes, and MS4A1 for B cells. Cluster-specific marker genes were identified using the FindAllMarkers function in the Seurat package, and the accuracy of cell annotation was further verified by referencing previously published human immune cell atlases.

To identify cluster-specific genes and screen differentially expressed genes (DEGs) among distinct cell subtypes, pairwise differential expression analysis was performed by comparing the gene expression level of each cluster against all other cells. The differential expression test was conducted using the sSeq method based on negative binomial exact tests for low read counts, while the asymptotic beta test embedded in edgeR was adopted for high read counts to ensure analytical efficiency and accuracy (16, 17). DEGs between the disease and control groups in each cell subtype were further validated using the MAST algorithm, a statistical method optimized for the dropout characteristics of single-cell transcriptomic data. Genes with an absolute log_2_ fold change (log_2_FC) ≥ 0.25 and Benjamini–Hochberg adjusted p-value (FDR) < 0.05 were defined as significantly differentially expressed. Volcano plots and heatmaps were generated to visualize the expression patterns of representative DEGs. Notably, CD14^+^monocytes isolated from pediatric gout patients exhibited significant upregulation ofIL1B and CXCL2 alongside downregulation of LYZ, indicating aberrant activation of the innate immune response in gout pathogenesis.

To systematically explore the biological functions and potential signaling pathways of the identified DEGs, Gene Ontology (GO) Biological Process (BP) and Kyoto Encyclopedia of Genes and Genomes (KEGG) pathway enrichment analyses were carried out using the clusterProfiler R package (v4.8.1) (18). Gene length bias was corrected during GO enrichment analysis, and GO terms with corrected P values < 0.05 were regarded as significantly enriched. The KEGG 2021 database was adopted for pathway annotation to systematically interpret the high-level biological functions and signaling pathways of marker genes derived from high-throughput sequencing data. Terms and pathways with adjusted p-values < 0.05 were considered statistically significant. Enrichment results revealed that the pathogenesis of pediatric gout was closely associated with multiple biological processes and signaling pathways, including NF-κB-mediated TNF signaling, endocytosis, autophagy regulation, and histone modification.

### Ethics approval and consent to participate

Informed consents were obtained from all children and their parents, and the study was approved by the Research Ethics Committee of Guangzhou First People’s Hospital, South China University of Technology, China(K-2024-129-02). Both the Declaration of Helsinki and the Good Clinical Practice Guidelines were followed and informed consent granted by all participants.

## Results

### Selective rewiring of the circulating immune interactome in pediatric gout

**Figure 1:**
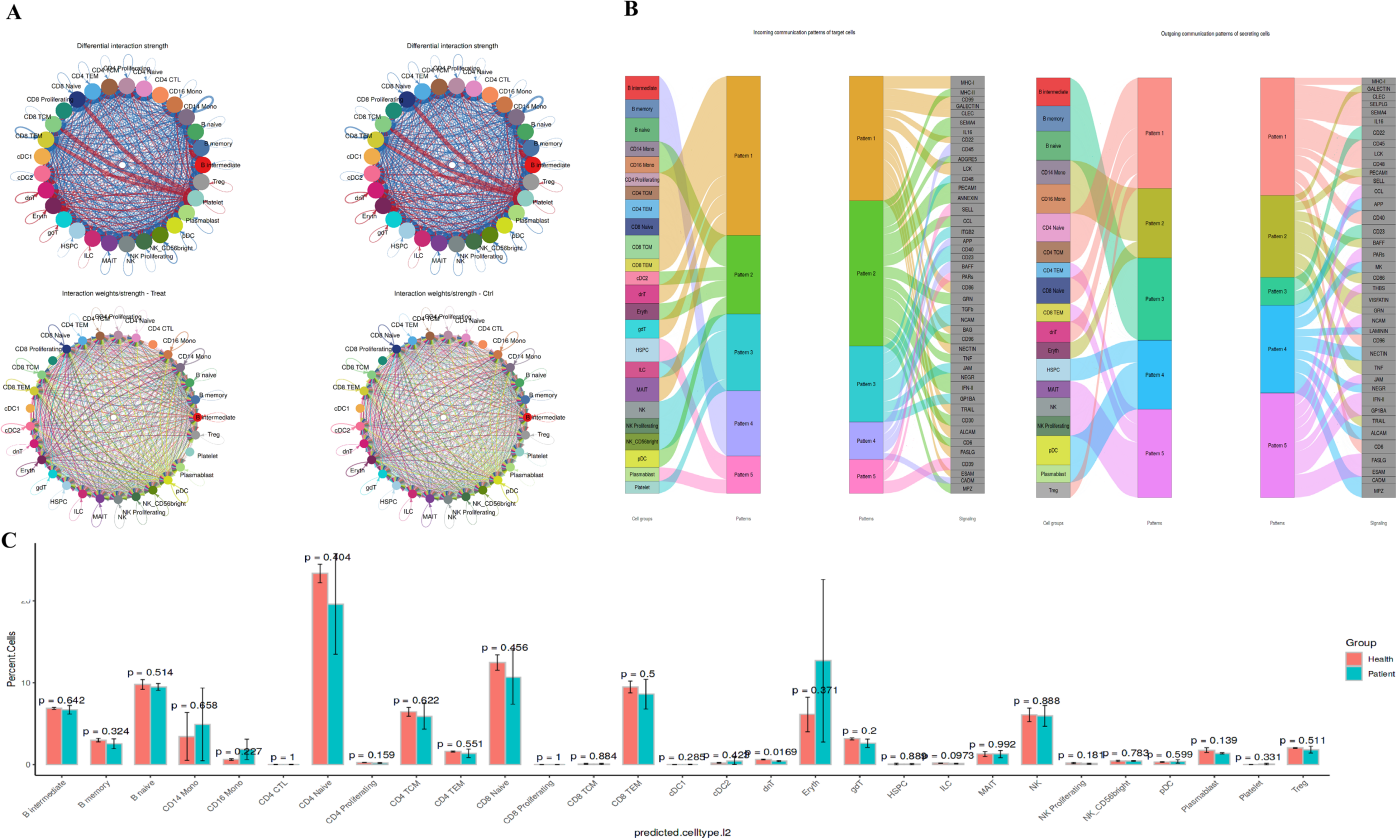
Single-cell atlas and global communication remodeling in pediatric gout. **(A)** Circular plots visualizing the global intercellular communication network. The patient group (Treat) exhibits a substantially denser and more complex network topology compared to controls (Ctrl), indicating widespread network remodeling. **(B)** Sankey diagrams depicting the outgoing (left) and incoming (right) communication patterns. The flow width represents the contribution of each cell type to specific communication modules, revealing a redistribution of signaling roles in the disease state. **(C)** Bar plot showing the proportional composition of circulating immune cell subsets in healthy controls (Ctrl) and pediatric gout patients (Treat). No significant differences in major cell populations were observed, excluding cell abundance as a confounding factor for subsequent communication analyses.

To determine whether pediatric gout arises from generalized immune hyperactivity or a structured reorganization of cellular crosstalk, we reconstructed the circulating immune interactome at single-cell resolution. While cellular composition remained comparable between patients and healthy controls (**Figures 1A, C**), the inferred communication network in pediatric gout exhibited a marked increase in density and complexity (**Figure 1B**). This expansion was not uniform; instead, it reflected a selective reconfiguration of specific signaling axes, indicating that disease-associated inflammation is an emergent property of a reorganized communication architecture rather than indiscriminate inflammatory amplification.

### Redistribution of sender and receiver identities underlies network remodeling

**Figure 2:**
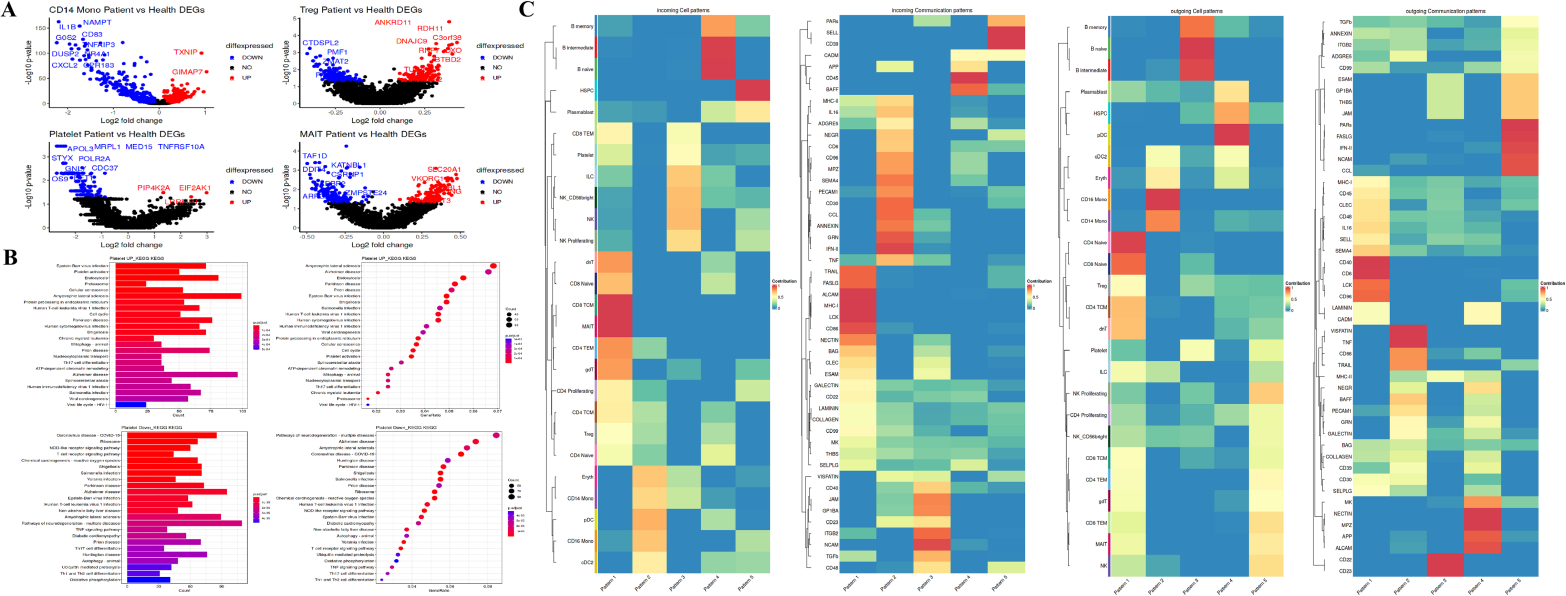
Sender-receiver analysis reveals platelet-to-monocyte communication dominance. **(A)** Dot plot showing specific cell-cell interaction strengths. The platelet-to-CD14+ monocyte interaction is significantly enhanced in pediatric gout, confirming a directional coupling between these populations. **(B)** Heatmap of outgoing communication patterns. Platelets show a striking gain in signaling output in the patient group (Treat), becoming the dominant sender population. **(C)** Heatmap of incoming communication patterns. CD14+ monocytes display the highest increase in signal reception in patients, positioning them as the primary receivers.

Having established large-scale communication remodeling, we next identified the cellular populations driving disease-associated signaling. This analysis revealed a pronounced reallocation of communication roles: platelets, which contributed minimally to the signaling network in controls, underwent a dramatic functional shift in pediatric gout, emerging as the dominant outgoing signaling population with extensive interactions across immune cell types. This transition positions platelets as active architects of the inflammatory communication network, not passive bystanders. Concomitantly, CD14+ monocytes exhibited the strongest increase in incoming signaling burden, establishing them as the principal recipient population in pediatric gout. The most prominent directional interaction occurred between platelets and CD14+ monocytes, indicating that this sender-receiver pair forms a central conduit for inflammatory signal propagation. Thus, the pediatric gout network is organized not by inflammatory cell expansion, but by directional coupling between newly activated signaling hubs and highly responsive recipient populations,as shown in **Figure 2**.

### ICAM1 enrichment in CD14+ monocytes anchors network asymmetry

**Figure 3:**
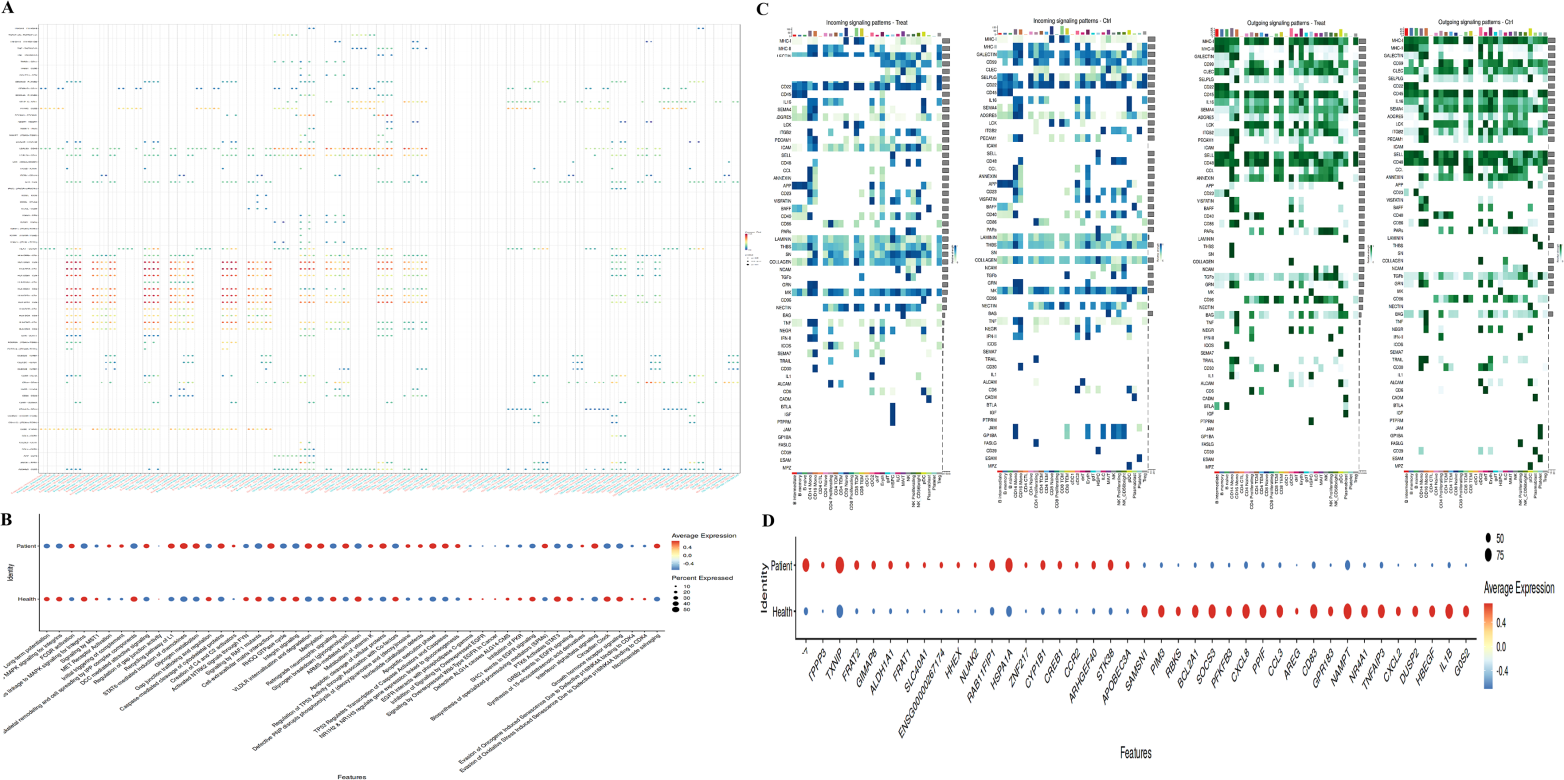
Pathway-level analysis identifies ICAM as the dominant disease-selective axis. **(A)** Heatmap comparing pathway activity between pediatric gout patients (Treat) and healthy controls (Ctrl). ICAM signaling exhibits the most pronounced and selective increase in patients (highlighted in red), whereas canonical immune recognition pathways (e.g., MHC-I, MHC-II) remain largely unchanged. **(B)** Dot plot showing ICAM1 expression across major immune cell populations. ICAM1 is specifically and significantly upregulated in CD14+ monocytes from the patient group (indicated by intense red dots), identifying them as the primary receivers in this signaling axis. **(C)** Volcano plot of differentially expressed genes in CD14+ monocytes. ICAM1 is highlighted as a top upregulated gene (red dot on the right), confirming its central role in the disease-specific transcriptional program. **(D)** Dot plot displaying the average expression and detection frequency of key inflammatory and metabolic genes. Red dots indicate high average expression in patient-derived cells supporting the transcriptional activation of pro-inflammatory pathways observed in the network analysis.

To link network architecture to molecular mechanism, we examined the ICAM signaling axis— the most significantly amplified pathway in pediatric gout. ICAM-mediated communication was negligible in controls but became a dominant signaling route in patients, both in absolute information flow and relative contribution to total network activity. We found that ICAM1 was selectively enriched in CD14+ monocytes, providing a mechanistic basis for their role as major signal receivers. This cellular enrichment of ICAM1 directly corresponded to enhanced ICAM pathway activity at the network level, establishing a clear link between transcriptional state and communication dynamics. Moreover, ICAM1 emerged as a top upregulated gene in CD14+ monocytes from pediatric gout patients, confirming its central role in the disease-specific transcriptional program. This molecular-network concordance solidifies ICAM signaling as a biologically coherent axis underlying inflammatory reprogramming in pediatric gout, as shown in **Figure 3**.

## Discussion

Pediatric gout has long been interpreted within the broader framework of urate-driven inflammation, yet its cellular organization has remained poorly resolved (19, 20). In this study, single-cell transcriptomic profiling combined with cell-cell communication analysis showed that the inflammatory state of pediatric gout is not defined by a uniform increase in immune activity, but by a selective rewiring of the circulating immune interactome. The most prominent feature of this remodeling was the emergence of ICAM signaling as a dominant disease-associated pathway. This observation shifts the focus from isolated inflammatory mediators to the organization of inflammatory information flow across cell populations. In this sense, pediatric gout appears to be shaped not only by urate-triggered immune activation, but also by a disease-specific communication architecture that structures how inflammation is propagated and sustained.

This perspective extends prior single-cell studies of gout while also clarifying what may be distinctive about the pediatric setting. Previous work in adult gout has emphasized inflammatory activation of monocytes, dysregulation of T cell compartments, and persistent immune remodeling across flare and remission (21–23). More recent pediatric single-cell studies likewise highlighted inflammatory transcriptional programs in CD14+ monocytes (24). Our findings are consistent with these observations, but add an additional layer of interpretation. Rather than identifying monocytes simply as activated inflammatory cells, we place them within a broader network context in which disease is defined by altered communication topology. The central question, therefore, is no longer only which cells are activated, but how signaling relationships among cells are reorganized in disease. Viewed from this angle, pediatric gout is better understood as a disorder of immune communication superimposed on canonical urate-associated inflammation.

A particularly striking aspect of this reorganization was the redistribution of signaling roles between platelets and CD14+ monocytes. In controls, platelets contributed little to the inferred communication network; in pediatric gout, however, they became the predominant outgoing signaling population. At the same time, CD14+ monocytes emerged as the major recipients of incoming signals. This sender-receiver asymmetry suggests that the inflammatory landscape of pediatric gout is organized through directional coupling rather than diffuse activation. The prominence of the platelet-to-monocyte axis is especially noteworthy because it expands the conventional monocyte-centered view of gout pathogenesis. Platelets are increasingly recognized as immune-active cells capable of shaping leukocyte behavior, and platelet-monocyte interactions are now understood to be important mediators of thromboinflammation and systemic immune activation (25–29). Our data therefore suggest that, in pediatric gout, platelets may function not merely as bystanders or secondary amplifiers, but as active hubs that help structure inflammatory propagation.

Among all inferred pathways, ICAM signaling showed the clearest and most disease-selective gain, and this network-level change was supported by selective enrichment of ICAM1 in CD14+ monocytes. This convergence between communication analysis and gene expression strengthens the view that ICAM1 is more than a passive marker of inflammation. Instead, it may act as a molecular scaffold that stabilizes and amplifies pathogenic intercellular interactions. This interpretation is consistent with broader work showing that ICAM-1 regulates leukocyte adhesion, inflammatory signaling, and tissue responses across multiple disease settings (30–32). The fact that MHC-I, MHC-II, and CD99 signaling remained comparatively stable further argues against a model of indiscriminate immune escalation and supports a more selective process centered on specific inflammatory circuits. From a translational perspective, this is important. Current treatment strategies for gout are largely directed at urate lowering and suppression of downstream inflammatory mediators. Our findings raise the possibility that, at least in a subset of pediatric patients, therapeutic benefit might also be achieved by disrupting the communication networks that sustain inflammation. In that framework, the ICAM axis emerges as a plausible candidate for network-directed intervention.

Despite the novel findings revealed by single-cell transcriptomic and cell-cell communication analyses, this study has several inherent limitations. First, the sample size is relatively small, including only three pediatric gout patients and three healthy controls. Although single-cell RNA sequencing provides high-dimensional transcriptomic data at the cellular level, the limited biological replicates may reduce the statistical power and generalizability of cell-cell communication inference and pathway enrichment analyses. Pediatric gout is a rare clinical disease, and it is difficult to enroll a large number of well-matched clinical samples in a single-center study. Future multicenter studies with expanded independent cohorts are required to validate our conclusions. In addition, our core findings including enhanced platelet-to-CD14^+^ monocyte communication and specific ICAM1 upregulation in CD14^+^ monocytes need to be further validated by orthogonal experimental approaches. Flow cytometry detection in a larger independent pediatric gout cohort will be performed in our follow-up work to verify the expression level of ICAM1 in CD14^+^ monocytes and the abnormal interaction between platelets and monocytes, which will provide solid experimental evidence for the ICAM-centered inflammatory network remodeling in pediatric gout.

## Data Availability

The datasets presented in this study can be found in online repositories. The names of the repository/repositories and accession number(s) can be found below: OMIX016700 (https://ngdc.cncb.ac.cn/omix/release/OMIX016700).
